# Over ten year follow-up results of a prospective and consecutive series of primary total knee arthroplasty with a multimodular total knee prosthesis

**DOI:** 10.1007/s00264-025-06634-w

**Published:** 2025-09-30

**Authors:** Claude Schwartz, Didier Mainard, Laurent Vastel, Jacques Hummer, Nicolas Hummer

**Affiliations:** 1 Colmar, France; 2 University of Lorraine, Nancy, France; 3 Raymond Poincaré University Hospital, Garches, France

**Keywords:** Total knee replacement, Multi-modular total knee replacement, Long term follow-up

## Abstract

**Purposed:**

High survival rates up to ten years have been reported for total knee replacements (TKR). Publications beyond ten years have more diverse conclusions. To study the long-term survival and clinical results of a multi-modular TKR, we examined a series of 120 cases with a minimum ten-years follow-up.

**Methods:**

This is a prospective multicentric observational study operated for arthritis by five senior surgeons. No control group has been included. Different femoral components (posterior stabilized (PS) or cruciate retaining (PC) versions), different tibial baseplates, both in cemented or cementless versions, were associated with an PS, PC or rotating version (PR) polyethylene insert and a patellar resurfacing or a partial inlay polyethylene component. In some cases the patella was not implanted. At ten years 38 patients were deceased (31.7 %) and 25 patients were lost to follow-up (20.8 %). 57 patients were reviewed at ten years.

**Results:**

The Kaplan Meier cumulative survival rate of the TKR, by considering revision for any reason as endpoint, at ten years is at 92.6% (CI: 86.2-96.1).They were two infections (1.7%) and eight other revisions for different reasons (6.7 %). Secondary IKS scores were statistically improved. Functional score was 79.4 points (p<0.001) and knee score was 89.2 (p<0.001). Minimal clinically important difference (MCID) were obtained in most of patients. Satisfaction of the patients was obtained in 93.75 % in a correspondence questionnaire.

**Conclusion:**

At ten year follow-up, this multi-modular TKR provides very good implant survival rates and clinical score with a high patient satisfaction.

## Introduction

High survival rates up to 10 years have been reported for total knee replacements (TKR), more for the femoral than for tibia components [1–6]. Publications beyond ten years have more diverse conclusions about the survival rates of these implants [8–14]. To study the long-term survival rates, safety and performance of a multi- modular total knee prosthesis, we examined a series of 120 TKR, with a minimum ten year follow-up.

The primary endpoint is evaluated by determining the proportion of patients undergoing prosthetic revision, along with the corresponding 95% confidence interval, at a ten year follow- up period.

The second objective of this study is to evaluate the clinical safety and the clinical performance of the medical device.

## Materiel and methods

This is a prospective multicentric observational study of 120 patients operated for primary or secondary knee osteoarthritis between April 2010 (first inclusion) and December 2011 (final inclusion), by five senior surgeons used to this TKR surgery.

It is a multicentric clinical study carried out in patients who have had the TKR fitted, in which its impact on safety and performance (Minimal Clinically Important Difference: MCID) is measured with a ten year follow-up. The MCID is the smallest change in a treatment outcome that a patient would identify as such [15, 16]. The MCID is an important concept used to determine whether an intervention improves perceived patient outcomes. The National Institute for Health and Care Excellence (NICE) guidelines provide information regarding this MCID. (https://www.nice.org.uk)

The outcomes were expressed as the percentage of patients attaining this clinical improvement. The threshold was taken as a minimal improvement of 9 for the knee score and a minimal of 10 for the function score.

### Population

The mean age of the population was 69.7 years (SD = 9.8; range: 43–90).

The population includes a majority of female (63%). The operated side is equally divided between right and left (49.7–50.3%). The average weight of the patients was 83.3 kg with an SD of 16.6 (min 43, max 130). The mean height was 165 cm (SD = 8; range: 145–185). The average BMI was 30.4 (18 to 47.8) with an SD of 5.5.

Most of the overall population had an ASA 2 classification [17]. 

**Table:** 

ASA 1	23.6%
ASA 2	60.4%
ASA 3	16%
ASA 4	0

38 of them (31.7%) had died within ten years of the operation and 25 patients (20.8%) were lost to follow-up during this period.

### Material

The prosthetic implants are the FHK^®^ brand implants (FH Ortho, Heimsbrunn, France).

The femoral components, (7 sizes), are available in posterior-stabilized (PS) or cruciate-retaining (PC), both in cemented (AC) or cementless (SC) versions.

The tibial baseplates (7 sizes), are available in fixed (PF) or rotating bearing (PR), both in cemented (AC) or cementless (SC) versions.

These devices are made of chrome-cobalt alloys.

The cementless implants have macrostructures ensuring primary fixation and feature a two- layer coating made of a plasma spray deposit of porous titanium and an hydroxyl apatite coating for secondary fixation on the femoral component.

The tibial inserts made in ultra high molecular weight polyethylene (UHMWPE), (6 thickness from 10 to 20 mm, from 2 mm in 2 mm) are available in the fixed PS, PC and the PR version. There is an optional tibial stem (length 70 and 110 m and diameter 10 or 14 mm).

The patella component in UHMWPE exists in two versions: cemented resurfacing (sizes 30, 34 and 38 mm) and inlay insert (sizes 22 and 25 mm), with or without cement.

All the implants are supplied sterile (gamma irradiation).

This variety of prosthetic implants allows each surgeon to make an accurate choice based on anatomy, ligament condition and physiological age.

Thus, 38 cruciate retaining (31.7%), 48 posterior-stabilized prostheses (40%) and 34 rotating plates (28.3%) were implanted.

Of the 120 TKR, 77 had a patellar resurfacing (64.2%), 31 an implant inlay (25.8%) and 12 (10%) had no patella prosthesis.

97 baseplates were cemented (80.8%), 64 of which had the femoral component also cemented (53.3% of the whole series); 53 femoral components were implanted without cement (44.2%) of which 20 cementless baseplate too (16.7% of the whole series); in three cases the tibia is cementless and the femur cemented.

The gestures associated with the surgery were: 48 partial synovectomy (40%), 30 medial release (25%), three lateral release (2,5%) and one anterior tibial tuberosity osteotomy (0,8%).

### Methods

The cumulative recovery rate (survival rate) was calculated using the Kaplan Meier method. Statistical analyses were performed using EasyMedStat. (www.easymedstat.com)

No replacement of missing data was performed in order to provide unbiased analysis.

Clinical performance’s assessments were measured using IKS scores [18]:

- Data describing the pain, the stability and the mobility of the knee: IKS knee score /100.
- Data describing the functionality of the knee through the activities specific to each patient: IKS function score/100.
- We also analysed subjective patient satisfaction with a questionary at 10 years which asked them if they were very satisfied, satisfied or disappointed with having this knee prosthesis procedure.

Numerical variables were expressed by their mean (± standard deviation, minimum, maximum) and discrete variables by their absolute and relative frequencies.

Quantitative variables will be described using the number of patients, arithmetic mean, standard deviation (SD), minimum, maximum.

Comparisons of pre- and postoperative quantitative data will be made with paired data tests (Wilcoxon signed-rank test) and a significance level of 5%. In case of low amount of patients, the normality will be verified with a Shapiro-Wilk test.

Comparisons of pre- and postoperative quantitative data was assessed with the Student’s paired t-test. Alpha risk was set to 5% (α = 0.05).

Evolution between pre and postoperative visits were made with paired data tests (Wilcoxon signed-rank test); A *p*-value < 0.05 reveal a significant change between the visits. In case of low amount of patients, the normality was verified with a Shapiro-Wilk test.

### Compliance with ethical standards

IRB approval: this is a non-interventional research not involving the human person (RNIPH); the information and non-opposition of patients as well as the processing of personal data has been operated in accordance with the Data Protection Act No. 2018-493 of May 3, 2018, and the General Data Protection Regulation (GDPR), applying the reference methodology MR-004. However, the study has been submitted on the Health data Hub (HDH 20221219135705).

### Conflicts of interest

The authors declare that they received funds because the licensed patent to FH Ortho, but they received no remuneration for this study. 

## Results

### Early complications

No intraoperative implant adverse event had occurred.

Two medically treated haematomas (1.7 %) cured without reintervention in 4 weeks.

### Late complications

One knee was mobilized under general anaesthesia for stiffness at two months, without revision of the implant, with a good final functional result.

Two infections (1.7%) were found. The first occurred 8 months postoperatively and the second more than two years following the intervention. Both were revised with implants changes and evolved without sequelae other than a loss of mobility about 20°in flexion.

Two patella dislocations occurred at short term (15 days and 6 months). The first one was in very obese patient with an important valgus knee. The second patient was 83 years old. A revision with transposition of the anterior tibial tuberosity in the first case and a section of the lateral retinaculum in the second case solved the problem.

Pain was the most frequent complication (4 cases; 3.3%), most often felt at the patella. Two were not labelled but still revised with prosthesis change, but without improvement.

Three complex regional pain syndrome (2.5%) occurred without obvious cause, and have conventional treatments (analgesics, neuropathic drugs as gabapentin, amitriptyline), mild and prolonged physiotherapy, and psychological care evolved very slowly towards recovery.

Three fractures (2.5%), following real traumas, were appeared in long term postoperative period (3 years, 8 years and 9 years). The first, a supracondylar fracture, was not treated because the patient was bedridden. The second, supracondylar, was treated using osteosynthesis. The third, inter and supracondylar fracture, was treated with a total revision of the prosthesis. These events were considered not directly related to the implants.

Thus, there were 7.5% of reoperations in total (*n* = 9).

The primary endpoint is assessed by calculating the proportion of patients with prosthetic revision and its 95% confidence interval.

### Revision rate and survivorship of the implants

**Fig. 1:**
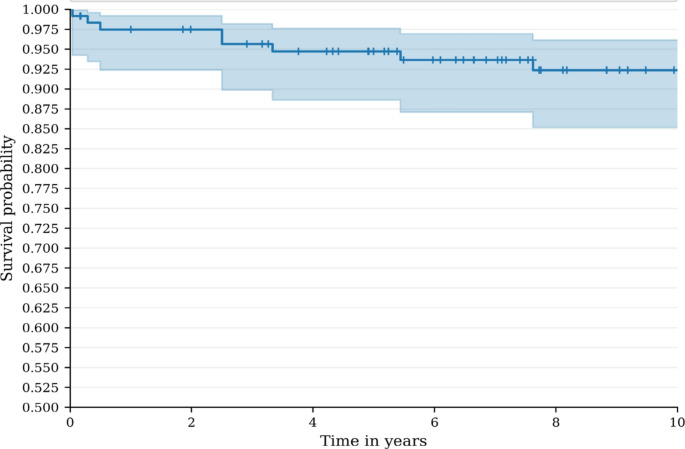
Kaplan-Meier cumulative survival rates by considering revision for any reason as endpoint for the overall population

Minimum follow-up time was 10 years. The Kaplan Meier cumulative survival rate, by considering revision for any reason as endpoint, at 10 years is estimated at 92.5% (95% CI: 86.2-96.1). 

**Table:** 

Delay	Survival	Confidence Interval
1 year	97.5%	92.4%-99.2%
2 years	97.5%	92.4%-99.2%
3 years	95.6%	89.9%-98.2%
4 years	94.7%	88.6%-97.6%
6 years	93.6%	87.1%-96.9%
8 years	92.5%	85.2%-96.1%
10 years	92.5%	85.2%-96.1%

### Clinical scores: IKS total score

#### Analysis of the overall population (Table 1)

The function IKS score of the overall population significantly improved (*p*-value < 0.001) from a preoperative mean score at 50.8 (SD = 20.6; range: 0 - 90) to a postoperative mean score at 81.9 (SD = 25; range: 0 - 100) at five years of follow-up and 79,4 (SD = 26,7; range: 0 - 100) at ten years. (p<0.001; significant p<0.05)

For the IKS Knee score, the comparison was statistically improved with a preoperative score of 27.9 (SD = 19.8; range: 0 - 78) to the five years postoperative follow-up with a mean score about 93.3 (SD = 4.6; range: 87 - 98) and 89,2 (SD = 12,5; range: 59 - 100) at ten years. (p<0.001; significant p<0.05).

**Table 1:** IKS scores – means function and knee scores – significance and MCID for all patients

IKS part	Parameters	Preop	Postop 5 years	Postop 10 years
Function	Mean – SD N=– [min- max]	50.8 ± 20.6 *n* = 120 [0; 90]	81.9 ± 25 *n* = 95 [0; 100]	79,4 ± 26,7 *n* = 59 [0; 100]
	Differences - Significance		*p* < 0.001*	*p* < 0.001*
	MCID (> 10)		74.8%	67,8%
Knee	Mean – SD N=– [min – Max]	27.9 ± 19,8 *n* = 120 [0; 78]	93,3 ± 4,6 - *n* = 95 [87; 98]	89,2 ± 12,5 *n* = 42 [59; 100]
	Differences - Significance	-	*p* = 0.002*	*p* < 0.001*
	MCID (> 9)	-	100%	100,0%

*: statistically significant (p<0.05)

All scores functional and knee scores were significantly improved. MCID were mainly attained.

#### Analysis according to the type of implants

The Table 2 is showing outcomes of the IKS Knee score depending of implants.

**Table 2:** IKS Knee scores according to the type of prosthesis

Type of prosthesis	Parameters	Preop	Postop 5 years	Postop 10 years
PF PC	Mean – SD N= [min – Max]	38 ± 17.5 *n* = 38 [0; 78]	93.3 ± 4.6 *n* = 14 [87; 98]	91.7 ± 15.7 *n* = 9 [63; 100]
	Differences - Significance	-	*p* = 0.048*	*p* = 0.001*
	MCID	-	85.3%	83,3%
PF PS	Mean – SD N= [min – Max]	21.3 ± 17.9 *n* = 48 [0; 67]	ND	91.8 ± 8.5 *N* = 18 [75; 100]
	Differences - Significance	-		*p* < 0.001*
	MCID	-		81,5%
PR PR	Mean – SD N= [min – Max]	18.5 ± 12.2 *n* = 34 [0;42]	ND	84.5 ± 13.9 *n* = 15 [59; 100]
	Differences - Significance	-		*p* = 0.383 NS
	MCID	-		40,0%

ND: No available data

*: statistically significant (p<0.05); NS = non-significant

All comparisons were statistically improved compared with the preoperative period. MCID were mainly attained.

The Table 3 is showing outcomes of the IKS Function score depending of types of prosthesis

**Table 3:** IKS function scores according to the type of prosthesis

Type of prosthesis	Parameters	Preop	Postop 5 years	Postop 10 years
PF PC	Mean – SDN= [min – Max]	54,1 ± 18,6 n=38 [0; 90]	83.7 ± 22.3 *n=34 [0; 100]*	87.5 ± 17.1 *n=12 [50; 100]*
	Differences - Significance	-	*p* = 0.048*	*p* = 0.001*
	MCID	-	85.3%	83,3%
PF PS	Mean – SD N=– [min – Max]	42,8 ± 22,8 *n* = 48 [0; 80]	76.3 ± 31 *n* = 34 [0; 100]	86,7 ± 20,2 *n* = 27 [30; 100]
	Differences - Significance	-	*p* < 0.001*	*p* < 0.001*
	MCID	-	27/36 (75.0%)	22/27 (81,5%)
PR PR	Mean – SD N=– [min – Max]	58,2 ± 15.6 *n* = 34 [20; 90]	86.4 ± 18,8 *n* = 29 [45; 100]	64,8 ± 33,1 *n* = 20 [0; 100]
	Differences - Significance	-	*p* < 0.001*	*p* = 0.383 NS
	MCID	-	68.4%	40,0%

*: statistically significant (p<0.05); NS = non-significant

All comparisons were statistically improvement compared with the preoperative period except for ten years for the MCID of the PR PE were mainly attained.

The Table 4 is showing outcomes of the IKS Function score depending of types of patella.

**Table 4:** IKS function score according to the type of patella

Type of prosthesis	Parameters	Preop	Postop 5 years	Postop 10 years
Inlay patella	Mean – SDN=– [min –Max]	55,2 ± 15,3 n=31 [15; 80]	80,7 ± 25.6 n=27 [0; 100]	65,9 ± 32,1 n=17 [0; 100]
	Differences - Significance	-	P<0.001*	p=0.348
	MCID	-	19/21 (90.5%)	6/17 (35,3%)
Resurfacing patella	Mean – SDN=– [min –Max]	48,9 ± 23,2 n=77 [0; 90]	80.5 ± 26.6 n=58 [0; 100]	84,4 ± 23 n=35 [15; 100]
	Differences - Significance	-	P<0.001*	p<0.001*
	MCID	-	42/52 (80.8%)	28/35 (80,0%)
No patella	Mean – SDN=– [min –Max]	51.3 ± 13.5 n=12 [35; 80]	91.3 ± 10.9 n=12 [70; 100]	87,1 ± 19,8 n=7 [50; 100]
	Differences - Significance		P<0.001*	p=0.016*
	MCID		46/56 (82.1%)	6/7 (85,7%)

*: statistically significant (p<0.05)

All comparisons were statistically improved compared to the preoperative period except for the inlay patella, probably due to the low number of patients (10 years 95% CI: 50,6- 81,1). MCID were mainly attained.

#### Analysis according to the type of fixation’s combinations

The following Table 5 is showing outcomes of the IKS function score depending of methods of fixations of tibial and femoral implants.

**Table 5:** IKS function scores according to the type of fixation’s combinations

Type of prosthesis	Parameters	Preop	Postop 5 years	Postop 10 years
Femoral AC – Tibial AC	Mean – SD N=– [min – Max]	47,2 ± 22,9 *N* = 62 [0; 90]	78,1 ± 28 *N* = 45 [0; 100]	76,9 ± 28,3 *N* = 31 [0; 100]
	Differences - Significance	-	*p* < 0.001*	*p* < 0.001*
	MCID	-	35/45 (77.8%)	20/31 (64,5%)
Femoral AC – Tibial SC	Mean – SD N=– [min – Max]	65 ± 18 *N* = 3 [45; 80]	45 ± 0 *N* = 2 [45; 45]	50 ± 70,7 *N* = 2 [0; 100]
	Differences - Significance	-	NPSA	NPSA
	MCID	-		
Femoral SC – Tibial SC	Mean – SD N=– [min – Max]	53,7 ± 14,7 *N* = 19 [20; 80]	88,8 ± 16,4 *N* = 17 [50; 100]	79 ± 22,5 *N* = 10 [35; 100]
	Differences - Significance	-	*p* < 0.001*	*p* = 0.039*
	MCID	-	14/17 (82.4%)	6/10 (60,0%)
Femoral SC – Tibial AC	Mean – SD N=– [min – Max]	54,6 ± 19,1 – *N* = 34 [0; 90]	85,6 ± 23,1 – *N* = 31 [0; 100]	90 ± 14,7 *N* = 14 [60; 100]
	Differences - Significance	-	*p* < 0.001*	*p* < 0.001*
	MCID	-	27/31 (87,1%)	12/14 (85,7%)

*: statistically significant (p<0.05); NPSA: No possible statistical analysis

 All comparisons were statistically improvement compared to the preoperative period. MCID were mainly attained. 

#### IKS pain sub-scores

The pain IKS score of the overall population significantly improved from 10.8 (SD = 13.2; range: 0 - 45) to 45.4 (SD = 10.2; range: 0 - 50) at five years of follow-up and 46,9 (SD = 7,7; range: 10 - 50) at ten years. 

The pain IKS score of the different evaluated subgroups also significantly improved (*p*-value < 0.001) at five years of follow-up and at ten years of follow-up, both for the type of prosthesis (Table 6) and the type of the patellar implant (Table 7).

**Table 6:** IKS Pain score according to the type of prosthesis

Type of arthroplasty	Preop	Postop 5 years	Postop 10 years	Significance
	Mean (SD) *N* =[Min; Max]	Mean (SD) *N* = [Min; Max]	Mean (SD) *N* = [Min; Max]	*P* Value preop- 5Y / *P* Value preop- 10Y
Overall	10,8 ± 12,3	45,4 ± 9,3	47 ± 7,6	*p* < 0.001*
population	*N* = 120 [0;45]	*N* = 95 [0; 50]	*N* = 57 [10; 50]	*p* < 0.001*
PF PC	16,4 ± 12,8	47,1 ± 7,3	48,8 ± 2,2	*p* < 0.001*
	*N* = 38 [0; 45]	*N* = 34 [20; 50]	*N* = 27 [45; 50]	*p* < 0.001*
PF PS	5,5 ± 10,8	45,3 ± 9,3	49,8 ± 0,9	*p* < 0.001*
	*N* = 48 [0; 45]	*N* = 36 [0; 50]	*N* = 28 [45; 50]	*p* < 0.001*
PR PE	10,2 ± 11,9	45,5 ± 10,5	41,6 ± 11,8	*p* < 0.001*
	*N* = 34 [0; 45]	*N* = 29 [0; 50]	*N* = 20 [10; 50]	*p* < 0.001*

*: statistically significant (*p* < 0.005)

**Table 7:** IKS pain score according to the type of patella

Type of Patella	Preop	Postop 5 years	Postop 10 years	Significance
	Mean (SD) *N* =[Min; Max]	Mean (SD) *N* = [Min; Max]	Mean (SD) *N* = [Min; Max]	*P* Value preop- 5Y *P* Value preop- 10Y
Overall	10,2 ± 12,3	45,9 ± 9,3	47 ± 7,6	*p* < 0.001*
population	*N* = 120 [0; 45]	*N* = 95 [0; 50]	*N* = 57 [10; 50]	*p* < 0.001*
Inlay	10,3 ± 12,4	43,3 ± 13,8	40,9 ± 12,3	*p* < 0.001*
patella	*N* = 31 [0; 45]	*N* = 27 [0; 50]	*N* = 17 [10; 50]	*p* < 0.001*
Resurfacing	9,8 ± 11,7	46 ± 9	49,5 ± 1,6	*p* < 0.001*
patella	*N* = 77 [0; 45]	*N* = 58 [0; 50]	*N* = 35 [45; 50]	*p* < 0.001*
No patella	10,6 ± 13,2	47,4 ± 5,1	49,2 ± 2	*p* < 0.001*
	*N* = 12 [0; 45]	*N* = 12 [20; 50]	*N* = 7 [45; 50]	*p* < 0.001*

*: statistically significant (*p* < 0.05)

At ten years there was no difference in IKS Pain score according to the type of combinations of fixation:

- Overall population: 47 ± 7,6 N =57 (10; 50) p<0.001*
- Femur and tibia cemented: 47,3 ± 7,8 N=31 (10; 50) p<0.001*
- Cementless femur and tibia: 45 ± 8,7 - n=9 [30; 50] p=0.02*

#### Questionary patient satisfaction

A total of 64 patients completed the ten year satisfaction questionnaire. Among these patients, 79.7% were “very satisfied” and 14.1% were “satisfied” after undergoing this surgery i.e. a satisfaction rate of 93.8%.

Considering different type of prosthesis, the satisfaction was 81% for PR PE and 100% for PF PC and PF PS.

Considering different type of patella’s, the satisfaction was 83.3% for inlay patella, 97.5% for resurfacing patella and 100% for no patella.

Considering different type of fixation, the satisfaction was 94.3% for Femoral AC -Tibial AC, and 100% for the other combinations.

### Discussion and conclusions

The 120 patients included in this study underwent TKR for first-line surgery. The reasons for this primary surgery include only for aetiology primary and secondary knee arthritis.

57 patients have a follow-up of at ten years. 64 people answered a questionnaire to assess their satisfaction with their expectations of the intervention.

The population includes a majority of female patients (63%).

The mean age at surgery (*n* = 120) was 69.7 years (SD = 9.8; range: 43–90). The average BMI was 30.4 (18 to 47.8) with an SD of 5.5.

In our series, there were not the main risk factors for instability, such as scleroderma, young age, phlebitis and stroke, as reported by Ergin and others [19]. 

We did not have any major deformations justifying a possible use of robotic techniques as reported by D Soundararajan et all [20]. 

The performance assessment is first based on the cumulative survival rate (Kaplan-Meier) and IKS knee and functional score between the preoperative period and the postoperative period performed with a follow-up of at least ten years.

We did not specifically investigate flexion mobility based on implant type (PS or medial pivot) such as B Obada [21], as this mobility is included in the knee section of the IKS score.

Considering revision for any reason as an endpoint, Kaplan-Meier’s analyses estimated a cumulative survival rate of the prosthesis at ten years (120 months) at 92.5% (95% CI: 86.2–96.1) considering the FHK prosthesis as a complete products line. The National Institute for Health and Care Excellence stated that total knee replacements are recommended as treatment options for people with knee arthritis if, and only if, the prostheses have revision rates of 5% or less at ten years. It is therefore possible to conclude that total knee replacement with FHK implantable medical devices is recommended as a treatment for osteoarthritis.

Our rate of postoperative complications requiring revision (other than fractures caused at late occurrence by trauma) was calculated at 5% with a 95% confidence interval at ten years. These are within the current limits recommended by NICE which is 5% maximum at ten years. To assess non-inferiority, the permitted upper confidence interval level is 10% underneath the benchmark standard which is 5.5% or less in 2024. (Australian Orthopaedic Association National Joint Replacement Registry Annual Report; https://aoanjrr.sahmri.com/annual-reports-2024).

The Swedish Knee Arthroplasty Register (https://sar.registercentrum.se/) has reported a ten year cumulative survival rate of approximately 95% too for total knee replacement (TKR) prostheses in 2023.

The United Kingdom Knee Arthroplasty Register (https://www.njrcentre.org.uk) has reported a ten year cumulative revision rate of approximately 5% too for total knee replacement (TKR) prostheses in 2022.

It is interesting to compare the results of our series with the data coming from the National Joint Registry (NJR) were the FHK prosthesis is entered since eleven years. In 2024, 1104 TKR were included with a mean follow up of 5,1 years. 16 TKR revisions were identified mainly due to infections (*n* = 6). Three adverse events occurred (0.19%).

The data of our series are in accordance with the independent data of the NJR. At ten years their cumulative revision rate is 2.2% (1.3%-5.1%). The cumulative revision rate of all other bicondylar TKR in the NJR is 3.0% (3.0%-3.1%) at ten years.

Given our cumulative ten-year revision rate of 5%, (late traumatic reasons excluded), it is therefore fair to say that the prosthesis used in our series is associated with a revision rate at least comparable, even a little lower, than some data from the world literature of high scientific level as exhaustive registers of several thousand cases *[Australia*,* England and Swedish mentioned above].*

The evaluation of results is then based on the data of our series are in accordance with final IKS “functional score” which improved significantly (*p*-value < 0.0001) from a mean preoperative score of 50.8 (SD = 20.6) to a mean postoperative score, over ten years, at 79.4 (SD = 26.7) and the final IKS “knee score” improved significantly too (*p*-value < 0.0001) from a mean preoperative score of 27.9 (SD = 19.8) to a mean postoperative score, at 89.2 (SD = 12.5) at ten years.

A total of satisfied patients were obtained in 60 cases out of 64 TKR at 10 years in both dimensions were obtained in most of patients. The clinically important minimum difference (MCID) is the smallest change in a treatment outcome that a patient would identify as such. This clinically felt minimal difference (MCID) is an important concept used to determine whether an intervention improves perceived patient outcomes. The National Institute for Health and Care Excellence (NICE) guidelines provide information regarding this MCID. (https://www.nice.org.uk)

In our study, this MCID for the IKS score was obtained for 67,8% of the population for IKS function score and 100% of patients for IKS knee score at control of 10 years compared to the preoperative state.

For all analyses and sub-analyses (depending on the type of implants), the Minimal Clinically Important Difference (MCID) [22–24] were presented. The threshold was taken as a minimal improvement of 9 for the knee score and a minimal of 10 for the function score. The outcomes were expressed as the percentage of patients attaining this clinical improvement. These results at 10 years are in line with the hypothesis formulated and with the best results of literature [25–28]. They will have to be confirmed by the continuation of the collection of clinical data.

However, this study has some limitations. These limitations are those of any study, retrospective or prospective: they are mainly missing data, especially the high rate of patients lost to follow- up at ten years and the rate of patients identified as deceased.

This deficit in the number of follow-up can have an impact on the calculation of survival rate and confidence intervals.

In addition, due to the absence of a control group and the absence of double blinding, the following methodological biases, classic and inherent in any study, must be taken into account: confounding bias (related to failure to account for confounders), monitoring and evaluation bias (related to lack of blinding and choice of outcomes potentially affected by patient autosuggestion and assessor subjectivity).

Due to the various constraints related to the evaluation of an implanted device, these biases are unfortunately still present in almost all publications in medical field.

However, despite the limitations mentioned above, our study can be considered to allow an evaluation of the different devices (FHK knee replacement) that is representative of current practice and thus, considering the safety results and functional scores presented above, it seems possible to conclude that these devices used in the placement of a TKR in our series are safe devices allowing functional improvement between pre- and post-operative states.

## Data Availability

Data is provided within the manuscript or supplementary information files.
